# Emergence of sparse coding, balance and decorrelation from a biologically-grounded spiking neural network model of learning in the primary visual cortex

**DOI:** 10.1371/journal.pcbi.1013644

**Published:** 2025-11-21

**Authors:** Marko A. Ruslim, Martin J. Spencer, Hinze Hogendoorn, Hamish Meffin, Yanbo Lian, Anthony N. Burkitt

**Affiliations:** 1 Department of Biomedical Engineering, University of Melbourne, Melbourne, Victoria, Australia; 2 School of Psychology and Counselling, Queensland University of Technology, Kelvin Grove, Queensland, Australia; 3 Graeme Clark Institute for Biomedical Engineering, University of Melbourne, Melbourne, Victoria, Australia; The University of Edinburgh, UNITED KINGDOM OF GREAT BRITAIN AND NORTHERN IRELAND

## Abstract

Many experimental and computational studies deal with sparseness, balance, and decorrelation in neural networks and explain the presence of these properties as fulfilling requirements related to optimum energy efficiency, network stability, and information representation. These studies leave the question of *how* these properties arise in the brain unanswered. The present study attempts to address this question using a model built upon the experimentally observed properties of neural responses, homeostasis, and synaptic plasticity. The experimentally observed properties of sparseness, balance, and decorrelation are then expected to emerge from this substrate. A spiking neural model of the primary visual cortex (V1) was investigated. Populations of both inhibitory and excitatory leaky integrate-and-fire neurons with recurrent connections were provided with spiking input from simulated ON and OFF neurons of the lateral geniculate nucleus. This network was provided with natural image stimuli as input. All synapses underwent learning using spike-timing-dependent plasticity learning rules. A homeostatic rule adjusted the weights and thresholds of each neuron based on target homeostatic spiking rates and mean synaptic input values. These experimentally grounded rules resulted in a number of the expected properties of information representation. The network showed a temporally sparse spike *response* to inputs and this was associated with a sparse *code* with Gabor-like receptive fields. The network was balanced at both slow and fast time scales; increased excitatory input was balanced by increased inhibition. This balance was associated with decorrelated firing that was observed as population sparseness. This population sparseness was both the cause and result of the decorrelation of receptive fields. These observed emergent properties (balance, temporal sparseness, population sparseness, and decorrelation) indicate that the network is implementing expected principles of information processing: efficient coding, information maximization (’infomax’), and a lateral or single-layer form of predictive coding. These emergent features of the network were shown to be robust to randomized jitter of the values of key simulation parameters.

## Author summary

Experimental evidence shows that visual information in the brain is represented using a neural code with particular features. First, individual neurons become selective and react strongly only to particular visual features. Second, neurons become diverse, representing different visual features. Third, neurons receive excitatory and inhibitory input that is balanced even over very short time scales (around 10 ms) inhibition and excitation rise and fall together. These three features are well known from experimental studies and are also well understood as providing an accurate and efficient code for visual information. Much less well understood is ***how*** these features arise.

In this paper we construct a simulation of ‘biologically grounded’ neurons in the primary visual cortex. Unlike the more familiar ‘Artificial Neural Networks’, biologically grounded neural networks are defined to have neurons that are only either excitatory or inhibitory in their effects on other neurons, and learning is achieved by neurons organizing themselves experimentally-based synaptic plasticity rules.

We observed that the accurate and efficient visual code emerged from the simpler rules of the biological neural network. This has implications for understanding the brain’s function and potentially also building more energy efficient machine learning.

## 1 Introduction

Neurons in the primary visual cortex are observed to show a **sparse response** to sensory stimuli; individual neurons respond strongly to only a minority of stimuli (temporal sparseness) ([1]). This sparse response of the cortex is associated with a **sparse code** that achieves an energy efficient representation of the input data, and maps the incoming sensory data to a small set of causes. These causes are the spatial receptive fields of each neuron; the particular visual input that causes a neuron to respond. In the visual cortex these receptive fields are experimentally observed to be well fitted by a Gabor function ([2]), a result that matches the theoretical predictions of sparse-coding models ([3–6])).

Biological networks also exhibit **balance** between excitation and inhibition ([7–9]). Balance is a dynamical feature of biological neural networks and is known to enforce stability and prevent runaway pathological responses ([10]). Balance can enhance the precision of cortical representations ([11]). The degree of balance can be evaluated at a range of timescales, from ‘loose’ balance for long time scales, to ‘tight’ balance for fine time scales ([12]). Loose balance refers to the overall magnitude of excitatory and inhibitory input to the neurons of the network. Tight balance can be observed in temporal correlations on the scale of milliseconds between the rapid changes in the excitation and inhibition provided to individual neurons.

**Decorrelation** in biological neural networks refers to de-correlated firing between individual neurons ([13]). This is a tendency for neurons to compete and reduce redundancy in the network’s representation of sensory input and maximise the information they carry about visual input. An individual visual stimuli results in activity in only a minority of neurons responding strongly, i.e., a form of ‘population sparseness’. Balance also appears to be associated with the decorrelation of responses between neurons; if inhibition balances excitation then the neuron will be prevented from firing. This creates a decorrelated spiking response which over time leads to decorrelation of the receptive fields of each neuron.

This literature shows that neurons in the visual cortex exhibit sparseness, balance, and decorrelation and offers valuable explanations for why these properties may be useful for energy efficiency, network stability, and optimum information representation. However, it does not answer the question of ***how*** the brain achieves these combination of features. To answer this question the present study takes a ‘bottom-up’ approach; a biologically grounded model was constructed based on experimental data of the neural dynamics and synaptic dynamics, and analysis of the results was used to determine whether the experimentally observed properties of neural representation emerge.

### 1.1 What are biologically grounded neural networks?

In biological networks neural activations (spike rates) cannot be negative, synaptic weights cannot switch sign ([14]), and synaptic weights are modified by local learning rules (rather than using a global optimization process like backpropagation). While there are many specific qualities that might be considered to be essential features of biological neural networks for the purposes of this study we define a biologically grounded neural network to have the following experimentally observed features related to the network’s architecture, neural dynamics and learning dynamics:

(i) Spikes used to transmit and encode information rather than scalar rate values often used in neural networks.
(ii) Distinct populations of excitatory and inhibitory neurons used rather than individual neurons that are simultaneously excitatory and inhibitory; a requirement known as Dale’s Law ([14]).
(iii) Specific to the visual cortex, input is provided from separate populations of ON and OFF Lateral Geniculate Nucleus (LGN) neurons encoding local positive and negative differences in luminance relative to background ([15]). This is more realistic than artificial neural networks that use negative inputs to represent below average luminance.
(iv) Lateral connections are included between neurons within a layer of the network. These are superfluous in networks that use back-propagation to create diversity.
(v) Spike-Timing-Dependent Plasticity (STDP) rules used to adjust synaptic weights based on the timing differences of spikes ([16]). These use local information available at the synapse rather than the explicit global objective functions and back-propagation used in artificial neural networks.

Biologically grounded neural networks can act as a biomimetic approach to improving Artificial Neural Networks (ANNs). In this context the model proposed in this study of a biologically grounded neural network can be thought of as a specific form of an Artificial Spiking Neural Network (ASNN) ([17]). Existing approaches to ANNs use an extremely large amount of energy during training ([18,19]). When implemented in neuromorphic hardware ASNNs are orders of magnitude more energy efficient ([20]). ASNNs are often trained using techniques such as a ‘shadow’ ANN ([21]), or back-propagation ([22,23]). While there has been moderate success using these methods non-biological, neither have matched the success of conventional ANNs and neither take full advantage of low-energy usage neuromorphic hardware.

Table 1 compares previous sparse coding models that learn information representation in V1 using some of the biologically-grounded features mentioned above and Table 2 compares the emergent properties observed in these models with the present study.

**Table 1:** Comparison of models of V1. Row headings are sparse coding models or models based on sparse coding and column headings are features of biologically-grounded neural models.

	LGN ON/OFF separation	Spiking (inputs, outputs)	STDP (feedforward, lateral)	Inhibitory subpopulation
[5,6,24]	✗	✗, ✗	✗, ✗	✗
[25,26]	✓	✗, ✗	✗, ✗	✗
[27,28]	✗	✗, ✓	✗, ✗	✗
[29]	✗	✗, ✓	✗, ✓	✓
[30]	✓	✓, ✓	✓, ✗	✗
[31]	✓	✓, ✓	✓, ✓	✗
Present study	✓	✓, ✓	✓, ✓	✓

**Table 2:** Comparison of the emergent properties of V1 models. Row headings are sparse coding models or models based on sparse coding and column headings are emergent features. Asterisks on the cited papers indicate those that also include an *n*_*x*_ vs *n*_*y*_ plot to illustrate the diversity of receptive field shapes.

	Sparseness		Balance	Decorrelation
	Imposed	Emerged	Analysed	
[24,25],[5]*; [6]; [26]*	✓		✗	✗
[27]*; [28]; [30]*; [31]		✓	✗	✗
[29]		✓	✗	✓
Present study*		✓	✓	✓

While many models of the visual cortex exist, there appears to be no model that combines biological realism, as defined above, with attempts to quantify the important properties of sparse coding (both spatial and temporal), balance, and decorrelation. For example, a recent model includes separate ON and OFF LGN inputs, spiking inputs and outputs, as well as STDP ([31]). However, this model does not include a separate inhibitory subpopulation and instead implements a single output population with recurrent inhibition. Additionally, this model does not demonstrate the diverse RF shapes seen in biology that other sparse coding models can account for ([5,26,27,29,30]). Another earlier model does show a variety of Gabor functions and includes a separate inhibitory subpopulation, but does not provide analysis of balance or decorrelation ([29]). It also omits the feedforward connection to inhibitory neurons and recurrent excitatory connections present in V1. Since these connections are known to exist in the visual cortex, it is necessary to show that their contribution is compatible with a sparse code.

### 1.2 Proposed model

**Fig 1:**
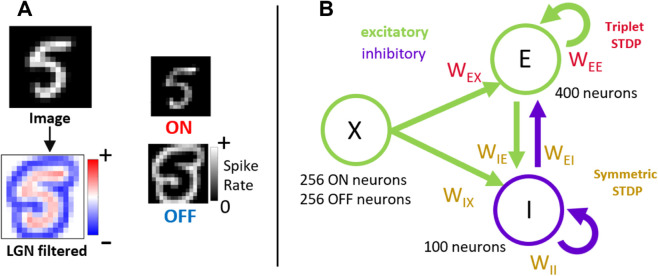
LGN model and cortical model topology: (A) LGN model. A grayscale image (16×16 pixels) spatially filtered using the center surround whitening filter. The filtered image pixel values converted into ON and OFF LGN neuron spike rates via rectification. (B) Cortical model: Separate populations of 400 excitatory and 100 inhibitory neurons received feed-forward input from 512 LGN neurons representing 256 image pixels. Synaptic connections with the inhibitory neurons were trained using symmetric STDP and connections between excitatory neurons were trained using triplet STDP.

The model in the present study consisted of V1 neurons driven by LGN neurons responding to visual stimuli. The LGN neurons were modeled as linear non-linear Poisson neurons, providing spike trains based on visual stimuli. To obtain the LGN neuron spike rates, natural images were spatially filtered (Fig 1A). This was achieved using the center-surrounding profile observed biologically ([32,33]).

Excitatory and inhibitory cortical neurons in the model were implemented as leaky integrate-and-fire (LIF) neurons (Fig 1B). The LIF model is a spiking neuron model that can accurately capture many of the most salient properties of neuron in the brain, such as the temporal integration of synaptic inputs and a non-linear (or threshold) firing mechanism ([34,35]).

The model used the triplet spike-timing-dependent plasticity rule for connections between excitatory neurons (Fig 1B). Triplet STDP is able to better capture observed plasticity in cortical neurons compared to classical pair-based STDP ([36]). Triplet STDP is mathematically equivalent to the Bienenstock Cooper Munro (BCM) learning rule for rate-based neurons ([36–38]) which has been influential in explaining several visual phenomena, including orientation selectivity, ocular dominance and monocular deprivation effects ([39]). For inhibitory synapses, a symmetric STDP rule was used ([40]) (Fig 1B). This rule corresponds closely to standard rate-based Hebbian learning and has been observed experimentally for inhibitory synapses of mouse auditory cortex ([41]).

The network incorporated separate excitatory and inhibitory populations, with a ratio of 4:1. This is consistent with cortical anatomy ([42]). Inhibitory neurons were set to have higher firing rates and stronger average output weights than excitatory neurons consistent with experimental observations ([43–45]).

Receptive fields of this model were examined to understand the selectivity of learned responses. The receptive field refers to the ideal sensory stimulus that will trigger the firing of a neuron. Classically, the receptive field is measured in experiments by presenting visual stimuli, such as spots of light, bars or gratings, and measuring the neuron’s response. White noise stimulation has also been used as the visual stimuli and this provides an unbiased estimate of the receptive field ([46,47]). Properties of the visual neuron can be inferred from their receptive field such as orientation and spatial frequency tuning. In this study, the shape and diversity of learned receptive fields were characterized, along with metrics such as sparseness, decorrelation and excitatory-inhibitory balance. Lastly, robustness of the network was tested by varying parameters such as target firing rates and weight jitter.

## 2 Methods

### 2.1 Network architecture

The visual stimuli to the thalamic neurons were provided by images with 16×16=256 pixels.

A population (X) of $N^{X}=512$ Lateral Geniculate Nucleus (LGN) neurons was split into 256 ON and 256 OFF sub-populations ([15]).

The cortical network simulated in this study consisted of $N^{E}=400$ excitatory (E) and $N^{I}=100$ inhibitory (I) neurons representing output V1 simple cells. Among these model V1 neurons there was all-to-all lateral connectivity with no self-connections.

All V1 neurons received feedforward thalamic input from all LGN neurons and all synapses were plastic and were trained during learning.

### 2.2 Image input and LGN processing

Raw images from the van Hateren dataset ([48]) were used to train the model. This dataset contains over 4000 black and white natural scene images with pixel intensities between 0 and 1. For these simulations, 1000 images were randomly chosen.

These images were first filtered using a centre-surround receptive field filter. This mimics the processing performed by the retina and LGN. The filter was a divisively normalised difference-of-Gaussians:

$$F(x,y)=\frac{F_{c}(x,y)-F_{s}(x,y)}{F_{d}(x,y)}\;,$$ (1)

where *F*(*x*, *y*) is the intensity at image location (*x*, *y*) after applying the spatial filtering, and $F_{c}$, $F_{s}$ and $F_{d}$ are normalized, concentric, isotropic Gaussian filters with standard deviations $\sigma_{c}=1$, $\sigma_{s}=1.5$, and $\sigma_{d}=1.5$, for center, surround and divisive normalization respectively. This is consistent with previous measurements ([32,33]). Images were then normalized to have a standard deviation of 1.

Random patches of size 16 × 16 pixels were sampled from these LGN-processed images, and selected as the input stimuli to the network.

Separate populations of ON and OFF LGN neurons were constructed by rectifying the positive and negative absolute values respectively (Fig 1A). Values were scaled to give appropriate firing rates across a large number of images. In particular image patches were scaled by a factor of $c_{r}=70$ to yield suitable firing rates (i.e., mean input firing rates of 20 Hz). Spikes were generated by simulating a Poisson processes. To simulate the saturation of retinal and LGN neurons, the Poisson firing rates of the LGN neurons were bounded at 100 Hz.

### 2.3 V1 neuron dynamics

Corrtical neural dynamics were simulated using a current-based LIF neuron ([49]). The governing dynamics for the membrane potential, $V_{j}^{A}(t)$, of neuron *j* in the output population were given by:

$$\frac{dV_{j}^{A}(t)}{dt}=\frac{V_{rest}-V_{j}^{A}(t)}{\tau_{m}}+\frac{I_{j}^{A}(t)}{C_{m}}\;,$$ (2)

where A∈{E,I} represents the excitatory and inhibitory populations, $C_{m}$ is the membrane capacitance, $\tau_{m}$ is the passive membrane time constant, and $V_{rest}$ is the resting membrane potential.

When the membrane potential crossed the spiking threshold, $\theta_{j}^{A}$, an action potential is generated and the membrane potential is reset to the resting potential. Additionally there was a lower bound for the membrane potential at $V_{min}$, analogous to the inhibitory reversal potential.

The synaptic input, $I_{j}^{A}(t)$, to neuron *j* in neural population A was modeled as an instantaneous current injection (for notational convenience the membrane capacitance parameter, $C_{m}$, was henceforth absorbed into the scaling of the weight matrix, ${𝐖}^{AB}$, of the network):

$$I_{j}^{A}(t)=\sum_{B\in \{X,E,I\}}\sum_{i}^{N^{B}}W_{ji}^{AB}S_{i}^{B}(t)+\xi_{j}^{A}(t)\;.$$ (3)

The synaptic weight from presynaptic neuron $i=1,2,...,N^{B}$ in population B to postsynaptic neuron *j* in population A is $W_{ji}^{AB}$. The spike train, $S_{i}^{B}(t)$, of neuron *i* in population B is represented by the sum of Dirac delta functions at the spike times. Gaussian white noise ($\xi_{j}^{A}(t)$), mimicking additional inputs from other more distant cortical neurons was added to the current at every δt=1 ms timestep sampled from the distribution $𝒩\left(\mu_{\xi },{\sigma_{\xi }}^{2}\right)$, where $\mu_{\xi }=0$ and $\sigma_{\xi }=\delta t=1$. The value of $\sigma_{\xi }$ was chosen to increase stochasticity of output spikes.

### 2.4 STDP and homeostasis

#### 2.4.1 STDP.

The form of STDP was chosen to depend on the type of synapse. For the synapses between two excitatory neurons (${𝐖}^{EX}$ and ${𝐖}^{EE}$) the minimal triplet STDP rule was used ([36]). Learning followed the spiking of postsynaptic spikes (at time *t*_*j*_) or the presynaptic neuron (at time *t*_*i*_):

$$\Delta W_{ji}^{EB}=\eta^{EB}A_{p}U_{p,i}(t)U_{a,j}(t-\epsilon ),\;if\;t=t_{j}\;\Delta W_{ji}^{EB}=-\eta^{EB}A_{d}U_{d,j}(t),\;\;\;\;if\;t=t_{i}\;,$$ (4)

where $W_{ji}^{EB}$ represents the synaptic weight from neuron *i* in population B∈{X,E} to neuron *j*, $\eta^{EB}$ is the learning rate and *ϵ* is a small positive constant to ensure that the weight change is updated before the synaptic trace variable, $U_{\alpha ,i}(t)$, is updated. The variables $U_{p,i}(t)$ and $U_{d,j}(t)$ are synaptic traces of presynaptic and postsynaptic activity, respectively. In general, these traces can be written as $U_{\alpha ,i}(t)$, where α∈{p,d} corresponds to the potentiation (p) or depression (d) component of the triplet STDP rule. Each synaptic trace was the convolution of a truncated exponential kernel, *K*_*α*_(*t*), with the neuron’s spike train *S*_*i*_(*t*):


$$K_{\alpha }(t)=ℋ(t)\exp\left(\frac{-t}{\tau_{\alpha }}\right),$$

$$U_{\alpha ,i}(t)=\left(K_{\alpha }*S_{i}\right)(t)\;,$$ (5)

where ℋ(t) is the Heaviside step function and $\tau_{\alpha }$ are the STDP time constants for α∈{p,d,a}. The STDP amplitudes are $A_{p}$ and $A_{d}$, which are the potentiation and depression sides of the triplet STDP respectively. The STDP time constants are $\tau_{p}$ and $\tau_{d}$. The third STDP time constant, $\tau_{a}$, is longer so that the resulting trace tracks an approximation of the moving average of the postsynaptic spike rate.

The threshold between potentiation and depression can be described by *ϕ* ([36,37]):

$$\phi =\frac{A_{d}\tau_{d}}{A_{p}\tau_{p}\tau_{a}}.\;$$ (6)

The ratio of the potentiation and depression coefficient values, $A_{p}$ and $A_{d}$, were chosen such that $\phi =\rho^{E}$, which is the target firing rate of the excitatory neurons. Setting the triplet STDP parameters in this way achieves rate equilibrium for uncorrelated input and output spikes. In the original BCM rule, the threshold varies to stabilize learning, and although the threshold in this triplet rule is fixed, the adaptive spiking threshold and weight normalization are expected to create stabilization.

For all other synapses (${𝐖}^{EI}$, ${𝐖}^{IX}$, ${𝐖}^{IE}$, and ${𝐖}^{II}$) the plasticity rule is symmetrical STDP. The symmetrical STDP rule was in the form:

$$\Delta W_{ji}^{AB}=\eta^{AB}A_{sym}U_{sym,i}(t),\;if\;t=t_{j}\;\Delta W_{ji}^{AB}=\eta^{AB}A_{sym}U_{sym,j}(t),\;if\;t=t_{i},$$ (7)

which has an STDP time constant of $\tau_{sym}$. This symmetrical STDP learning rule applied to synapses from population B∈{E,I,X} to population A∈{E,I}, except the external-to-excitatory (X→E) and reccurent excitatory-to-excitatory (E → E) connections, which follow the triplet STDP rule described in Eq 4.

#### 2.4.2 Weight normalization.

All synaptic weights to a neuron had an L1 norm upper bound. If the L1 norm is exceeded due to STDP during training, then those weights are normalized using first subtractive normalization followed by synaptic weight bounding and then multiplicative normalization.

For example, $W_{j\cdot }^{AB}$, which is the weights from population B∈{E,I,X} to postsynaptic neuron *j* in population A∈{E,I}, underwent subtractive normalization if the L1 norm exceeds $l_{1}^{AB}$:

$$W_{j\cdot }^{AB}\to W_{j\cdot }^{AB}-\frac{\|W_{j\cdot }^{AB}{\|}_{1}-l_{1}^{AB}}{N^{B}},$$ (8)

where $l_{1}^{AB}$ is the upper bound of the L1 norm and $N^{B}$ is the number of neurons in population B. Weights were then constrained to be non-negative: $W_{ji}^{AB}\to \max(W_{ji}^{AB},0)$ to enforce Dale’s law leading to an increase in $\|W_{j\cdot }^{AB}{\|}_{1}$. Multiplicative normalization was then applied:

$$W_{j\cdot }^{AB}\to W_{j\cdot }^{AB}\times \frac{l_{1}^{AB}}{\|W_{j\cdot }^{AB}{\|}_{1}}.$$ (9)

Subtractive normalization was applied (in addition to multiplicative normalization) to avoid the weight-convergence that occurs under multiplicative normalization when there is a large growth in the mean synaptic weight.

#### 2.4.3 Homeostatic plasticity.

Homeostatic plasticity was also employed in the form of an adaptive spiking threshold, $\theta_{j}^{A}$, for neuron *j* in population A={E,I} ([27,50]):

$$\Delta \theta_{j}^{A}=\eta_{\theta }^{A}\left[\frac{1}{T}\int_{0}^{T}S_{j}^{A}(t)dt-\rho^{A}\right],$$ (10)

where $\eta_{\theta }^{A}$ is the homeostatic learning rate, *T* is the learning period and $S_{j}^{A}(t)$ is the spike train. This homeostatic rule ensured the excitatory (inhibitory) neurons have a time-average firing rate of $\rho^{E}$ (*ρ*^I^). An upper bound on $\Delta \theta_{j}^{A}$ was enforced to avoid instability.

### 2.5 Synaptic balance in the network

Balanced network theory was used to determine appropriate mean weights and inform the choice of L1 upper bounds of synaptic weights. The balance theory condition under large *N*, which is the total number of neurons in the network, is that the mean total synaptic current to a neuron scales as *O*(1). Balance and a stable solution is obtained if:

$$\frac{{\bar{w}}^{EX}}{{\bar{w}}^{IX}}>\frac{{\bar{w}}^{EI}}{{\bar{w}}^{II}}>\frac{{\bar{w}}^{EE}}{{\bar{w}}^{EI}},$$ (11)

where ${\bar{w}}^{AB}$ is the mean synaptic weight from neurons in population B∈{E,I,X} to neurons in population A∈{E,I}. This relation is similar to the balanced condition derived in other studies ([51–53]).

$$\rho^{I}=2\rho^{E},$$ (12)

$${\bar{w}}^{\cdot I}\approx 2{\bar{w}}^{\cdot E},$$ (13)

where ${\bar{w}}^{\cdot E}$ (${\bar{w}}^{\cdot I}$) is the mean synaptic weight with an excitatory (inhibitory) presynaptic neuron. These parameter choices are grounded in experimental findings: inhibitory interneurons tend to fire at higher rates than excitatory neurons in vivo ([43]), and inhibitory synapses are often stronger and more reliable than local excitatory synapses ([44,45]).

The values of the L1 weight norm upper bounds were chosen to adhere to Eqs 11 and 13 and are shown in Table 3. Eqs 12 and 13 are enforced by the adaptive spiking threshold (Eq 10) and homeostatic weight normalization (Eqs 8 and 9).

**Table 3:** Simulation parameter summary for the network model.

Neural Populations		
**Name**	**Value**	**Description**
$N^{X}$	512	Size of ON and OFF input population, X: 16×16×2
$N^{E}$	400	Size of excitatory population, E
$N^{I}$	100	Size of inhibitory population, I: $0.25N^{E}$
**LGN Processing**		
**Name**	**Value**	**Description**
$\sigma_{c}$	1	Standard deviation of center Gaussian filter
$\sigma_{s}$	1.5	Standard deviation of surround Gaussian filter
$\sigma_{d}$	1.5	Standard deviation of divisive normalization Gaussian filter
$c_{r}$	70	Scaling constant to yield suitable LGN firing rates after LGN processing
**Neuron Model**		
**Name**	**Value**	**Description**
$\tau_{m}$	10 ms	Membrane time constant
$V_{rest}$	0	Resting and reset membrane potential
$V_{min}$	-10	Lower bound of membrane potential
$\theta_{j}^{\alpha }$	10 (initial)	Adaptive spiking threshold for an excitatory neuron (α=E) or an inhibitory neuron (α=I)
δt	1 ms	Simulation time step
$\sigma_{\xi }$	1	Standard deviation of synaptic current Gaussian white noise
**Weight Connectivity**		
**Name**	**Value**	**Description**
$l_{1}^{EX}$	100	L1 norm upper bound of weights from X to E
$l_{1}^{EE}$	10	L1 norm upper bound of weights from E to E
$l_{1}^{EI}$	120	L1 norm upper bound of weights from I to E
$l_{1}^{IX}$	80	L1 norm upper bound of weights from I to X
$l_{1}^{IE}$	240	L1 norm upper bound of weights from I to E
$l_{1}^{II}$	120	L1 norm upper bound of weights from I to I
**Plasticity Model**		
**Name**	**Value**	**Description**
$\rho^{E}$	2 Hz	Target firing rate of the output excitatory neurons during image presentation
$\rho^{I}$	4 Hz	Target firing rate of the output inhibitory neurons during image presentation
$\tau_{p}$	20 ms	Time constant of potentiation for triplet STDP
$\tau_{a}$	50 ms	Long time constant of potentiation for triplet STDP
$A_{p}$	1	Coefficient of potentiation for triplet STDP
$\tau_{d}$	20 ms	Time constant of depression for triplet STDP
$A_{d}$	0.1	Coefficient of depression for triplet STDP: $\rho^{E}\tau_{a}$
$\tau_{sym}$	20 ms	Time constant of symmetrical STDP
$A_{sym}$	0.5	Coefficient for symmetrical STDP
$\eta^{EX}$	$2\times 10^{-4}$	Learning rate for weights from X to E
$\eta^{EE}$	$1\times 10^{-4}$	Learning rate for weights from E to E
$\eta^{EI}$	$9\times 10^{-2}$	Learning rate for weights from I to E
$\eta^{IX}$	$3\times 10^{-3}$	Learning rate for weights from X to I
$\eta^{IE}$	$4\times 10^{-2}$	Learning rate for weights from E to I
$\eta^{II}$	$6\times 10^{-2}$	Learning rate for weights from I to E
$\eta_{\theta }^{E}$	0.1	Homeostatic rate for adaptive spiking threshold of excitatory neurons
$\eta_{\theta }^{I}$	0.1	Homeostatic rate for adaptive spiking threshold of inhibitory neurons
**Stimulus**		
**Name**	**Value**	**Description**
$N_{n}$	1200	Number of epochs/batches
$N_{b}$	100	Number of image patches in a batch
*T*	0.4 s	Duration of one learning period and stimulus presentation

### 2.6 Training

The initial weight connectivity structure was random and sparse. Some weights were initialized to high values whereas others were initialized to low values:

$$W_{ji}^{AB}=\left\{ \begin{array}{cc} b & with\;probability\;p,\\ 0.01\;b & otherwise, \end{array}\right.$$ (14)

where *b* is sampled from a normal distribution with mean 1 and standard deviation 0.5, and *p* = 0.2 was the probability of a strong synaptic connection. Weights were then scaled to their upper bound L1 norm (Eq 9).

During training each image was presented to the network for *T* = 400 ms. Images were presented in batches of 100. The spiking thresholds were allowed to reach stable values by running the network with an adaptive spiking threshold but no weight plasticity for 100 batches (100×100×400 ms ≈67 mins in model time). After this period the weights and spiking threshold were both allowed to adapt.

Training occurred over *N*_*n*_ = 1200 batches (1200×100×400 ms ≈11.1 hrs model time). At both batch number $0.3N_{n}$ and batch number $0.6N_{n}$, the learning and homeostatic rates, $\eta^{AB}$ and $\eta_{\theta }^{A}$ were halved to facilitate both fast initial learning and finely tuned final synaptic weights.

### 2.7 Tabular summary of parameters

### 2.8 Analysis methods

#### 2.8.1 Receptive field analysis.

**Spike-Triggered Average** Following training, the receptive fields (RFs) of the modelled V1 cells were estimated by Spike-Triggered Average (STA) ([46]), also referred to as reverse correlation or white-noise analysis.

The STA provides an unbiased estimate of a neuron’s receptive field only if the stimulus distribution is spherically symmetric, e.g., Gaussian white noise ([46,47]). Spatial Gaussian white noise stimulus with unit variance, **n**, was put through the same LGN pipeline, scaled by a factor of 2$c_{r}$ to convert to spiking rate and presented to the network, and the firing rate, *r*, of each model cell recorded. Gaussian white noise was converted to suitable pixel values between 0 and 255 using the relation: 50𝐧  +  100, with lower and upper bounds of 0 and 255 respectively. The receptive field of each neuron, **F**, was estimated using a weighted average of the images:

$$𝐅=\frac{\sum_{i=1}^{N_{w}}r_{i}{𝐧}_{i}}{\sum_{i=1}^{N_{w}}r_{i}}\;,$$ (15)

where $N_{w}=10^{5}$ was the number of white noise stimuli. The results of the STA were upsampled from 16×16 pixels to 160×160 pixels using bilinear interpolation.

**Fitting Gabor-functions to receptive fields:** To facilitate comparison of simulation results with experimental data the RFs of the V1 neurons were then fit with Gabor filters.

The process used was similar to previous experimental ([2]) and simulation studies ([26]). A 2D Gabor function, *G*(*x*, *y*), is defined as a sinusoidal plane wave multipled by a 2D Gaussian window. As in [26], the fitting error is defined as the ratio of the sum of the squared residuals over that of the receptive field. The receptive fields that had a fitting error of less than 10% were described as well-fit to a Gabor function. The fitted $n_{x}=\sigma_{x}f_{s}$ and $n_{y}=\sigma_{y}f_{s}$ values give the width and length of the Gabor function expressed as the product of the standard deviations of the Gaussian envelope, $\sigma_{x}$ and $\sigma_{y}$, and the spatial frequency *f*_*s*_.

#### 2.8.2 Quantifying sparseness.

The modified Treves-Rolls sparseness metric was used to quantify **temporal** sparseness (sometimes called lifetime sparseness) for a given neuron ([54]):

$$\varsigma_{T}=1-\frac{(\sum_{j}^{M}r_{j}/M{)}^{2}}{\sum_{j}^{M}(r_{j}^{2}/M)}\;,$$ (16)

where *r*_*j*_ is the neuron’s response rate to image *j*, and *M* is the number of images used.

An identical metric was used to quantify the **population** sparseness in response to a given image:

$$\varsigma_{P}=1-\frac{(\sum_{i}^{N}r_{i}/N{)}^{2}}{\sum_{i}^{N}(r_{i}^{2}/N)}\;,$$ (17)

where *r*_*i*_ is each neuron *i*’s response rate to the image, and *N* is the number of neurons in the population.

The value of ς in each case lies between [0, 1] and approaches 1 for a highly sparse responses. To obtain a measure of the overall temporal and population sparseness the average value across neurons was used to characterize temporal sparseness ($\bar{\varsigma_{T}}$) and the average value across images was used to characterize population sparseness ($\bar{\varsigma_{P}}$).

#### 2.8.3 Network robustness.

To test robustness of the results to parameter changes, simulations were run in which the upper bounds for the L1 norm of the weights for E, I and X inputs were each randomly varied. For each simulation, each of these parameters was scaled by a random number sampled from the uniform distribution between 1–*c* and 1 + *c* for each postsynaptic neuron. For instance, $l_{1}^{AB}$ would become a $N^{A}\times 1$ vector where neurons in population A may have different weight norm upper bounds.

Simulation parameters remain the same (Table 3) except for the L1 norms which are jittered and the learning rates. Due to different L1 norms, the learning rates for each group of weights were adjusted to compensate. For example, for the weights from feedforward to excitatory neurons:

$$\eta_{new}^{EX}=\eta_{old}^{EX}\times \frac{l_{1,new}^{EX}}{l_{1,old}^{EX}}$$ (18)

#### 2.8.4 Varying target firing rates.

Simulations with different target firing rates were run. In particular, excitatory neurons had target firing rate of 1 Hz, 2 Hz, 5 Hz, 10 Hz or 20 Hz. The inhibitory neurons had a target firing rate of double that of the excitatory neurons, namely 2 Hz, 4 Hz, 10 Hz, 20 Hz and 40 Hz, respectively. This choice is consistent with experimental findings showing that neurons fire within this frequency range ([55]) and that inhibitory interneurons tend to fire at higher rates than excitatory neurons ([43]). Ten simulations for each of these target firing rates were run.

Simulation parameters remain the same (Table 3) except for the the depression coefficient of triplet STDP was required to satisfy equation 6. Additionally, due to different firing rates, the learning rates were adjusted to compensate. For example, for the weights from feedforward to excitatory neurons and for recurrent excitatory weights:


$$\eta_{new}^{EX}=\eta_{old}^{EX}\times \frac{\rho_{old}^{E}}{\rho_{new}^{E}}$$

$$\eta_{new}^{EE}=\eta_{old}^{EE}\times {\left(\frac{\rho_{old}^{E}}{\rho_{new}^{E}}\right)}^{2}$$ (19)

#### 2.8.5 Varying network size.

When varying the number of cortical neurons multiples of the total number of neurons in the original model were chosen: $c_{n}\in \left\{1,\;2,\;3,\;4\right\}$. For the input neurons, there were $c_{n}$ ON and OFF neurons per pixel, leading to $c_{n}N^{X}$ input neurons; for the output excitatory and inhibitory neurons, there were $c_{n}N^{E}$ and $c_{n}N^{I}$ neurons respectively.

The L1 norms were scaled in proportion to $\sqrt{N}$ to conform with known effects in vivo ([51–53]) and in vitro ([7]). Additionally, the learning rates were adjusted as in Eq 18.

## 3 Results

Before training, weights were initialized to their L1 upper bound. It was observed that when weights were initialized with very small non-zero values, the L1 weight norms all approached upper bound, and took much longer to learn (S1 Supplementary Material).

The baseline network firing in response to random background input before learning was observed to be maintained after learning (S2 Supplementary Material).

### 3.1 Sparseness in the network’s activity

**Fig 2:**
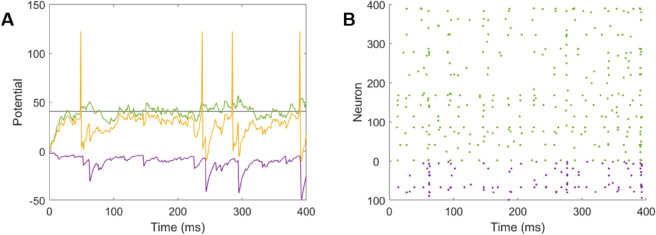
Membrane voltage and rastor plot after training: (A) The membrane voltage response of an excitatory neuron (E-388) in response to a single image presented for 400 ms after learning. Membrane voltage (yellow), black (spike threshold). The green and purple traces show the response to excitatory and inhibitory inputs alone. (B) The raster plot of the spiking response of all neurons in response to the same single image. The population sparseness is $\varsigma_{p}^{E}=0.90$ and $\varsigma_{p}^{I}=0.86$ for the excitatory (green) and inhibitory (purple) population respectively. All neural parameters as described in Table 3.

After training, the neurons show membrane voltage dynamics and spike patterns typical of biological networks (Fig 2A and 2B). The sparseness value of the excitatory neurons in this for the trained network of $\varsigma_{p}^{E}=0.90$.

**Fig 3:**
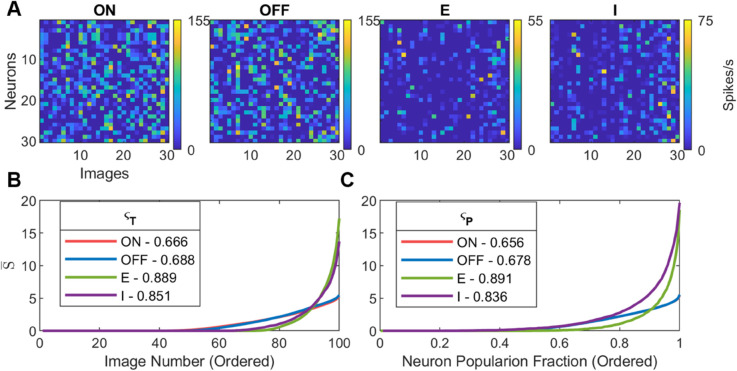
Sparse network response after training: (A) The spike rate of a subset of 30 neurons in each neural population (ON, OFF, E, and I) in response to a subset of 30 images. (B) Temporal Sparseness in response to 100 images. This is calculated by first sorting each neuron’s response (the rows of the arrays in (A)) from lowest to highest response spike rate before then averaging across rows to give mean spikes rate $\bar{S}$ Note that the red and blue traces are closely aligned. C) Population Sparseness. This is calculated by first sorting each image’s response (the columns of the arrays in (A)) from lowest to highest response spike rate before then averaging across columns. The horizontal axis shows all the neurons ordered from lowest to highest response for each images, and then normalized so that the sparseness curves can be compared directly despite differing population size for ON, OFF, E, and I. All neural parameters as described in Table 3.

Spike rates for each neuron were observed across multiple images (Fig 3A). Qualitatively, each neuron in each population appears to show temporal sparseness. This can be seen in the fact that each neuron responds selectively with a high spike rate produce only to certain images.

To quantify this effect each of the rows of the response matrices were ordered from lowest to highest response across the 100 images used, these ordered rows were then averaged together to give the populations average spike rate ($\bar{S}$) as a function of their least preferred to most preferred image (Fig 3B). The approximately exponential distribution of spike rates indicates that neurons were selective in their response, which is indicative of spareness. The value of temporal sparseness for each population, $\bar{\varsigma_{T}}$ was calculated using Eq 16. The values of $\bar{\varsigma_{T}}$ show that the E and I populations are more sparse than the ON and OFF populations and that the E population was more sparse than the I population.

Similar analysis was performed for populations sparseness (Fig 3C). The approximately exponential curve in Fig 3C can be interpreted as the average distribution of spike rates across the population in response to an image.

### 3.2 Sparseness in the network’s receptive fields

**Fig 4:**
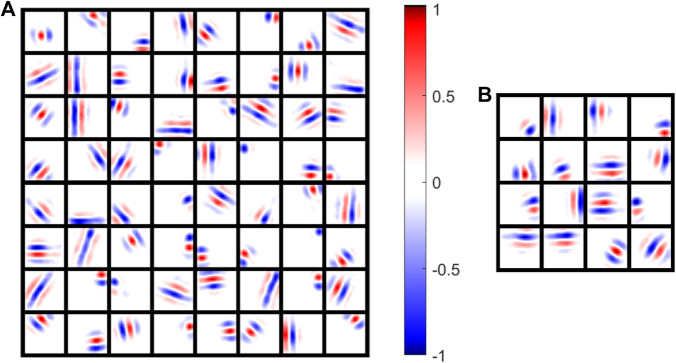
Receptive fields of excitatory and inhibitory neurons characterized via simulated STA: Weights calculated via STA have been normalized. (A) Excitatory receptive fields of 64 randomly chosen neurons. Red and blue shows the measured weight to pixels of the visual field represnting that neuron’s sensitivity to above average or below average luminance levels in that pixel. (B) Inhibitory receptive fields of 25 randomly chosen neurons. Neural parameters were those as described in Table 3.

The spike triggered average (STA) for each neuron in the E and I populations was calculated, as described in Sect 2.8.1, and representative receptive fields are shown in Fig 4. A diverse range of receptive field shapes were learned, such as localized unoriented blob-like filters and oriented Gabor-like filters. It can be seen that the network learned Gabor-like receptive fields, which also arise from other sparse coding models ([24,26,27,29]). Furthermore, receptive fields of different sizes, positions and orientations can be seen for both excitatory and inhibitory neurons.

**Fig 5:**
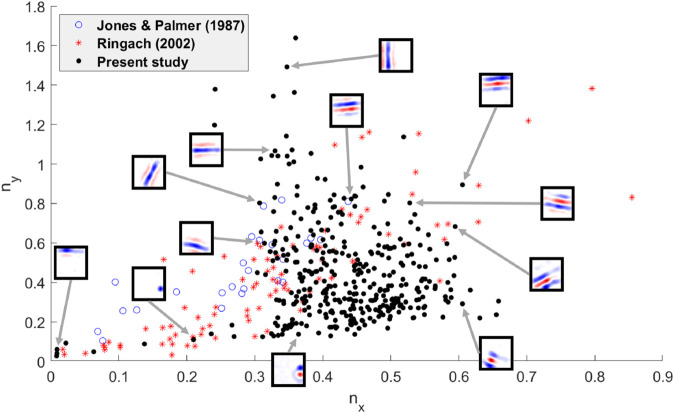
Quantified comparison of Gabor functions with experimental data ($n_{x}\;vs\;n_{y}$): Distribution of receptive fields of model excitatory neurons compared with experimentally recorded receptive fields for cat ([56]) and macaque monkey ([2]). Open blue circles: cat data. Red stars: monkey data. Black dots: model data of 369 of 400 excitatory neurons that fit Gabor filters with <10% fit error. Example RFs from the model are shown in inset.

Receptive fields were quantified by fitting each one to a parameterized Gabor function as described in Sect 2.8.1. The 369 out of 400 excitatory neurons that have a fit error less than 10% are shown with experimental data from cat and macaque (Fig 5). $n_{x}(=\sigma_{x}f_{s})$ and $n_{y}(=\sigma_{y}f_{s})$ are the width and length of the Gabor function expressed as the product of the standard deviations of the Gaussian envelope, $\sigma_{x}$ and $\sigma_{y}$, and the spatial frequency *f*_*s*_.

It can be seen that the Gabor functions are situated in the same region of parameter space as the Gabor functions measured from mammals *in vivo*. However, there are some regions in the *n*_*x*_, *n*_*y*_ space that is occupied by experimental results but not by the model.

### 3.3 Decorrelation in the network’s activity

**Fig 6:**
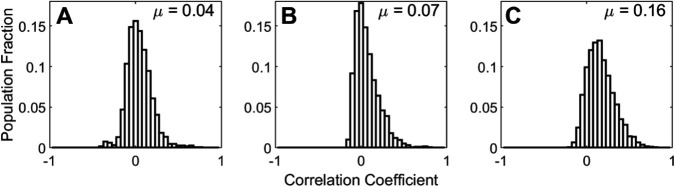
Correlation of firing rates using data from 100 images: (A) Pairwise correlation coefficients of LGN neurons across 100 images, (B) excitatory population, and (C) inhibitory population.

To examine de-correlation of firing rates the spike rate data across neurons and images shown in Fig 3A was used. The correlation coefficient between the firing rates of each pair of LGN, E, and I neurons was calculated (Fig 6).

It can be seen that the LGN and E populations have small mean correlation coefficients. The distributions are somewhat skewed indicating that highly correlated firing is more likely than highly uncorrelated firing.

### 3.4 Decorrelation in the network’s receptive fields

**Fig 7:**
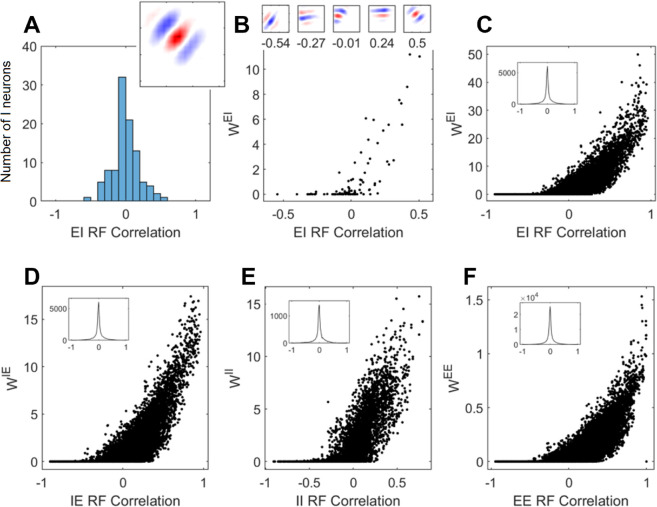
Decorrelation of receptive fields: (A) The range of correlations between an excitatory neuron’s receptive field (receptive field shown in inset) and the receptive fields of the 100 inhibitory neurons in the network. (B) The inhibitory input weight from each of the inhibitory neurons as a function of their receptive field correlation. Example inhibitory neuron receptive fields at different correlation values are shown in inset. (C-F) The input weight to as a function of the pairwise receptive field correlations for every EI neuron pair, IE neuron pair, II neuron pair and EE neuron pair. The insets show the histogram of the RF correlations using bins of width 0.02. Neural parameters as described in Table 3.

The correlation coefficient of a single excitatory neuron’s receptive field with each of the 100 inhibitory neuron’s receptive fields was calculated as described the the Methods giving a distribution of values (Fig 7A). The majority of the inhibitory neurons are uncorrelated (showing zero correlation) while a minority have higher correlated or anti-correlated values.

The pairwise correlation coefficient is also plotted for all 40,000 excitatory-inhibitory pairs (Fig 7C, inset). There is decorrelation between these two populations, with coefficients clustered at 0 and no apparent positive bias. The same analysis reveals similar uncorrelated receptive fields among all populations (Fig 7D-F, insets).

### 3.5 The mechanism of decorrelation in the network’s receptive fields

To assess the role of inhibitory interneurons in decorrelating the responses of primary (excitatory) neurons the inhibitory input weight from all inhibitory neurons was plotted as a function of the correlation coefficients between the pairs (Fig 7C). The same analysis was completed for all 40,000 pairs of inhibitory input to excitatory neurons in Fig 7D and for the reciprocal case of excitatory input to inhibitory neurons in Fig 7E.

It can be seen that excitatory neurons receive strong input from inhibitory neurons with strongly correlated receptive fields (Fig 7C). The data shown in Fig 7E and 7F shows a similar synaptic weight dependence on RF correlation for inhibitory-inhibitory and excitatory-excitatory connections.

### 3.6 Balance in the network’s activity

**Fig 8:**
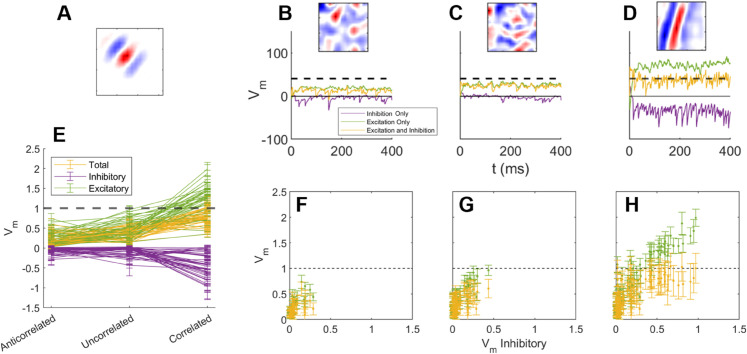
Loose balance: (A) An excitatory neuron’s receptive field as measured via STA. (B) The neuron’s membrane voltage during the network’s response to the anti-correlated image stimulus shown in the inset. The image was selected as the most anti-correlated from 1000 images. The spike-threshold and voltage minimum was removed to allow observation of the full inhibitory response. The response without inhibition and without excitation is shown in green and purple. (C) Response to the most uncorrelated image. (D) Response to the most correlated image. (E) Using the same approach as in (B-D), the average membrane voltage for 40 randomly chosen neurons in response to the most anti-correlated, un-correlated, and correlated images (chosen from 100 random images). Error bars show the standard deviation in the voltage. (F-H) The excitatory and total membrane membrane voltages plotted as a function of the amplitude of the inhibitory only voltage for using the data in (E). Neural parameters as described in Table 3.

To examine loose balance in the network an excitatory neuron was randomly selected and its receptive field characterised by STA (Fig 8A). To quantify the total excitatory and inhibitory input the neuron’s spike-threshold and minimum membrane voltage limit were both removed to allow the membrane voltage to evolve without interference. Anti-correlated (low-input), uncorrelated, and correlated (high-input) image patches were chosen from random set of 1000 patches. These three images provide the neuron with its lowest to highest input drive and are shown in Fig 8B-D (insets).

The membrane voltage during the network’s response to these images (Fig 8B-D (yellow trace)) shows an increase with increasing input as expected. The membrane voltage with only excitatory input and with only inhibitory input is also shown (Fig 8B-D, green and purple traces).

For this neuron across these three images it can be seen that the increase in excitation during the network’s response to the correlated image patch was accompanied by an increase in inhibition. This analysis was repeated for 40 randomly chosen neurons and the mean and standard deviation of the total (yellow), excitatory (green) and inhibitory (purple) membrane voltages calculated (Fig 8E). It can be observed that excitation and inhibition *both* grow with increasing input intensity. Despite increasing input intensity (anti-correlated, uncorrelated, correlated) the total membrane voltage (yellow) for all 40 neurons has a mean and standard deviation that rarely transits above the spike-threshold.

To examine the presence of balance in more detail, for these 40 neurons the mean excitatory input and combined input was replotted as a function of the mean inhibitory input (Fig 8F-H, green). The values can be seen to be correlated and that the mean membrane voltage (yellow) as a function of the mean inhibitory input shows that the total voltage is brought down below the spike-threshold. This appears to demonstrate that neurons are not excitation dominated, even at high input levels (Fig 8 H).

**Fig 9:**
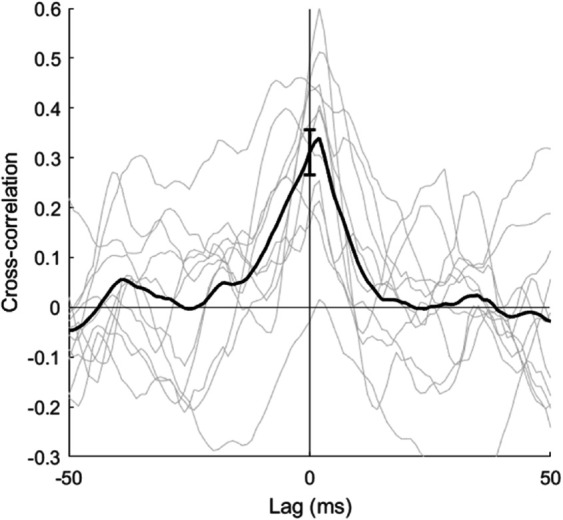
Tight balance: The cross correlation between the excitatory-only and inhibitory-only responses of the neuron in Fig 8A when input is provided by ten correlated images patches similar to that shown in Fig 8D (inset). The black trace shows the mean of the ten traces and the error bar gives the confidence interval of the mean at a lag of 0 ms.

The cross-correlation between the excitatory and inhibitory response to the ten high input (correlated) image patches was calculated (Fig 9). The period of response from 0 to 100 ms was excluded to avoid measurement of correlations in the initial transient change in membrane voltage. There was a positive temporal correlation between the excitatory and inhibitory inputs to the neuron. The correlation decays with a time-window of approximately 10 ms, a similar time-scale to that of the membrane time constant, $\tau_{m}$.

### 3.7 Robustness of the network

**Fig 10:**
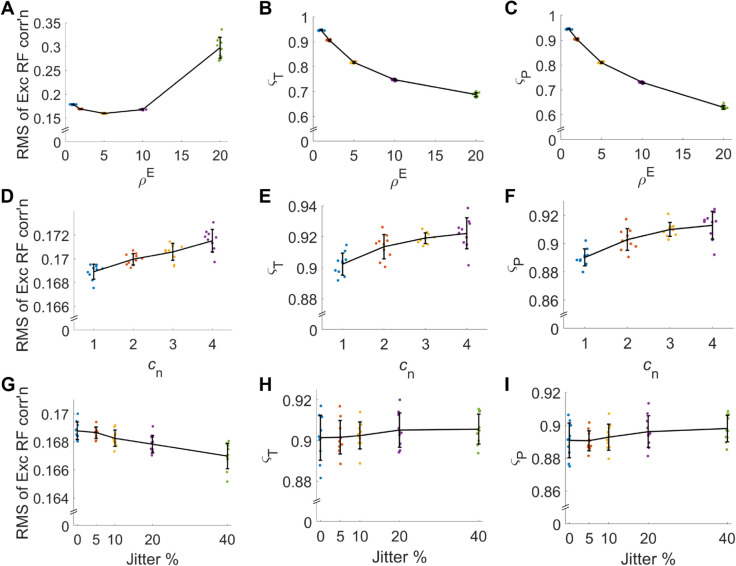
Robustness of the network: (A-C) Firing rates of excitatory and inhibitory neurons are varied. (D-F) Total number of neurons in the network are varied, where $c_{n}$ is the multiple of the default total number of neurons. (G-I) Mean weights are jittered. (A,D,G) RMS of the Pearson correlation of the receptive fields between all excitatory cell pairs. (B,E,H) Temporal sparseness, $\varsigma_{T}$, is measured. (C,F,I) Population sparseness, $\varsigma_{P}$, is measured. Neural parameters are varied as described in Methods 2.8.3 and 2.8.4. Other neural parameters as described in Table 3. Black lines connect the means, and errorbars display the standard deviation of individual simulations represented as coloured dots that have a random jitter in the x-axis for visibility.

The network’s sensitivity to excitatory neuron target firing rate, neuron number, and L1-norm values was examined by setting these values before training and then training the network. In response to each the change in excitatory receptive field correlation, temporal and population sparseness were quantified (Fig 10).

When their target firing rate was increased excitatory receptive fields became more correlated with each other (Fig 10A). There was also a decrease in temporal and population sparseness (Fig 10B and 10C). Analytic results show that similar effects are seen in an analytic single-neuron model (S3 Supplementary Material).

When the total number of neurons was increased, the excitatory receptive field correlation, temporal and population sparseness show a small increase (Fig 10D-F).

When the L1 weight norms are varied up to 40%, metrics such as excitatory receptive field correlation, temporal and population sparseness remained relatively constant (as shown in) Fig 10G-I).

## 4 Discussion

In this study a spiking network model of V1 was developed using a bottom-up approach based upon biological principles, including separate ON and OFF inputs, spiking neurons, separate excitatory and inhibitory populations (Dale’s Law), spike-timing-dependent plasticity, and firing rate homeostasis. It was found that after training the model exhibited several properties observed in biological systems: a sparse neural responses, decorrelation of activity and receptive fields, and balanced excitation and inhibition. Additionally, the network was robust to changes in network size as well as to random jitters to the synaptic weights, but not to high target firing rates of the output neurons.

### 4.1 Emergence of sparseness in the network

The sparse *temporal* spiking activity observed in Fig 3A shows that each neuron is selective, responding at high rates only to specific images. This appears to be similar to available experimental data ([1,57]). This qualitative assessment is quantified in Fig 3B-C. These values were however shown to be vulnerable to increases in target spike rate (Fig 10B). Using analysis of the LIF model (See: S3 Supplementary Material) this was discovered to be a feature of the LIF neural dynamics: without other changes, temporal sparseness decreases with increasing spike rate. It is unknown whether this is also a feature of biological cortical neural responses, but is a possible avenue for future experimental work.

As shown in Fig 10 temporal sparseness in a LIF model is affected by the spike rate with a lower spike-rate leading to a higher temporal sparseness. This may explain the lower temporal sparseness of the inhibitory population seen in Fig 3.

This temporal sparseness is known via mathematical sparse-coding models to be associated with learning of Gabor filters in the visual cortex ([3,24]). These Gabor filters are also found in the present model (Figs 4 and 5).

Experimental measures of population sparseness are difficult to obtain because they require simultaneous measurement from many cortical neurons. However the sparse *population* spiking activity observed in Fig 3A and quantified in Fig 3C appears to qualitatively match the available experimental data ([1]).

These collection of results indicate that the network has sparse properties without sparseness being achieved via an objective function or otherwise explicitly imposed. Instead, the LIF model with adaptive excitability established a sparse firing response to stimuli which provided correlations for the triplet-STDP rule to discover.

### 4.2 Emergence of decorrelation in the network

Fig 6 shows that the mean spike rate correlation coefficient was positive for the excitatory (Fig 6B) and inhibitory populations (Fig 6C). It is unknown whether this non-zero mean population decorrelation is a feature of biological cortical neurons and this perhaps is a question for experimental work. This appears to be an effect that is inherited and amplified from the LGN inputs which also showed a positive mean correlation coefficient (Fig 6A). LGN neurons are known to show a bias towards OFF features in natural scenes ([33]). The mean correlation coefficient between pairs of inhibitory neurons is higher. This increase in correlated firing for the I population is possibly due to the different feed-forward learning rules. The E population triplet rule discovers second order correlations in the inputs ([31]), while the symmetrical rule used for the inhibitory population discovers first order correlations.

The decorrelation of receptive fields within the E (Fig 7F inset) and I (Fig 7E inset) populations did not reflect this positive bias in spike response correlations. Instead the receptive fields were well de-correlated.

These results showing decorrelation of activity and receptive fields were again, not something that was imposed explicitly using an objective function. Instead, symmetric STDP rules in inhibitory neurons discovered correlated firing in excitatory neurons (Fig 7D). Effectively, they are gathering evidence that those excitatory neurons have similar receptive fields. Simultaneously, symmetric STDP rules in excitatory neurons discover correlated firing in inhibitory neurons (Fig 7E) gathering evidence of their similar receptive fields. Combined, this leads to indirect inhibition between excitatory neurons ([29]).

The fact that the inhibitory neurons received strong input from excitatory neurons with correlated RFs (Fig 7D) can be combined with the fact that excitatory neurons receive input from inhibitory neurons with correlated receptive fields (Fig 7C) to indicate that, effectively, excitatory neurons provide *inhibitory* input to other similar excitatory neurons via the inhibitory population. This is similar to the results in [29]. The resulting activity in the population of excitatory neurons will over time tend to avoid redundant representation of the visual input.

The data for excitatory-excitatory connections (Fig 7F) shows that there is a tendency for these weights to cause correlated activity in the network. In this network, the weights of excitatory to excitatory connections are low compared to other lateral weights, and so the effect is negligible. However, it is possible that this apparently detrimental mechanism has a beneficial role in temporal tasks where direction selective cells provide additional information, something not investigated here.

### 4.3 Emergence of balance in the network

Biological networks in the brain are known to exhibit balance in the contributions of excitation and inhibition to each neuron. The neuron in Fig 2A appears to be in a fluctuating regime ([58,59]), in which the membrane potential tends to sit just under the spiking threshold, and fluctuations cause irregular spiking activity. This appears to be due to a balance of excitation and inhibition (the green and yellow traces). To examine this, correlations in the magnitude of total excitatory and inhibitory input to excitatory neurons were observed in the trained network (Fig 8D, 8E, and 8H). These ensured that even with strong input, the neurons did not become excitation dominated. The fast temporal correlation on the time scale of ∼10 ms is an example of tight balance (Fig 9). It can be seen that the inhibitory input lags the excitatory input by approximately 2-3 ms. This delay is likely to be a combination of the extra synapse and the integration of inputs by the inhibitory neurons.

The total excitatory and inhibitory input to each neuron is decided by balance network theory using the inequalities in Eq 11 enforced via the homeostasis described by Eq 9. However, this is insufficient to ensure the resulting tuned and fine balance in Fig 9. Instead this balance has emerged as a consequence of a combination of the homeostasis and the synaptic learning rules that ensure that excitatory neurons receive input from inhibitory neurons with similar receptive fields.

Although balance theory was used as a guiding principle to set the upper bounds of the L1 weight norm, it is important to note that this balance condition was derived under specific conditions to apply the mean-field theory: namely that input neurons fire at a constant and identical firing rate, and that output neurons are independent due to the random structure of the weights. However, in our simulations, input neurons fire at different firing rates depending on the visual stimuli, and the weights in our network show structure due to learning.

### 4.4 Relationship between sparseness, decorrelation, and balance

Population sparseness in activity is partly the result of decorrelation due to lateral synaptic plasticity. The population sparseness in activity results in changes in the correlations discovered by the excitatory neurons among their feed-forward input weights from LGN neurons. Once these changes in receptive field have taken place, population sparseness in activity is also due simply to the decorrelation in receptive fields among the E neuron population. In this way population sparseness and decorrelation operate on the fast time scales of the network’s immediate response (∼10 ms) to inputs but also the slow time scales of synaptic plasticity (∼1 h).

This population sparseness in excitatory neuron receptive fields ensures that the network maximizes the information it carries about its inputs. This is something that an ‘infomax’ function would normally explicitly require to ensure statistical independence ([60,61]). In the biologically-grounded neural network used in this study, this decorrelation emerges from learning rules governing the connections to and from inhibitory neurons.

As highlighted above, the tight balance shown in Fig 9 is closely related to decorrelation. The correlated inhibitory inputs prevents the neuron from firing at times that are are predictable by other neurons in the network. This suppression of predictable responses is reminiscent of the principles of efficient coding ([62]), and predictive coding ([63]).

### 4.5 Comparison of receptive fields with experimental studies

The network model in this study has separate excitatory and inhibitory populations with all-to-all feedforward and recurrent connectivity that are learnt via STDP. When whitened images were provided to the LGN input neurons, the output neurons learned visual receptive fields that resembled the shape and diversity of receptive fields measured experimentally, as illustrated in Fig 5. In particular, localized un-oriented blob-like receptive fields as well as localized oriented Gabor-like receptive fields of varying sizes and spatial frequencies were observed to closely resemble those observed in physiological studies ([2]). Inhibitory neurons show a range of properties in experimental studies ([64]), including orientation tuning, and they have receptive fields that resemble those of excitatory cells ([65,66]). The inhibitory neurons in the network learned receptive fields that resemble those found in experimental studies.

While the model successfully produces a diversity of receptive field shapes, it does not exhibit an organized orientation preference map across the population of neurons, such as pinwheels or hypercolumn structures observed in the primary visual cortex of species like cats and primates ([67,68]). This is primarily because the model neurons do not have a topographic arrangement with distance-dependent lateral connectivity. In this current form, model neurons have all-to-all connectivity; introducing spatial connectivity constraints such as distance-dependent connectivity is a direction of future work.

The 31 cells that had RFs not well fitted by Gabor filters may be in a transition state from one RF to another RF or be blob-like RFs.

### 4.6 Network robustness to spike rate, neuron number, and L1-norms

Excitatory neurons show reduced temporal (Fig 10B) and population sparseness (Fig 10C) as well as an increase in pairwise RF correlation (Fig 10A). This is consistent with the neurons becoming less selective with increasing spike rate. This is something re-enforced by the analytic model results (S3 Supplementary Material).

The increase in temporal and population sparseness increase with neuron number (Fig 10D-E) appears to show that greater numbers of neurons more effectively create population decorrelation. Despite this, the RF correlation increased with the number of neurons (Fig 10A). This can potentially be explained as the result of more neurons representing the same visual space. Together, these results demonstrates that the network can scale up to larger neuron numbers seen physiologically.

Simulations with different mean weight values (Fig 10G-H) result in different amounts of excitatory and inhibitory input which could potentially cause an excitatory-inhibitory imbalance. However, all simulations conducted have resulted in stable learning and activity. One reason is the adaptive spiking threshold, which is adjusted to ensure that neurons fire within stable levels. As well as remaining stable, certain metrics remain at similar values despite changes in the weight norms. This includes the pairwise excitatory receptive field correlation, temporal and population sparseness.

### 4.7 Spike timing dependent plasticity

The synaptic plasticity rules used in the study presented here are those that have been observed experimentally. In particular, the type of STDP implemented depends upon the identity of the presynaptic and postsynaptic neuron. Synapses where the presynaptic and postsynaptic neuron are both excitatory neurons learn via the STDP triplet rule ([36]). This rule fits experimental data well and can be mapped to the BCM rule in rate-based neural models, which has been used to model visual phenomena ([39]). A recent spiking network used the triplet rule to also produce visual receptive fields and showed that using the triplet rule allowed their neurons to ignore low-order correlations and find features hidden in higher-order statistics ([31]). Their model, however, implements an output population only with one output population with recurrent inhibition, instead of separate excitatory and inhibitory subpopulations. In our model, inhibitory synapses learn through symmetrical STDP. Experimental studies have observed that the order of spike times (pre-before-post or vice versa) does not affect inhibitory plasticity ([41]). Moreover, symmetrical STDP can be mapped to rate-based Hebbian learning ([40], Supplementary Material). The results here indicate that symmetrical STDP, because of its associative Hebbian-like learning, is sufficient to find correlations between the output excitatory and inhibitory neurons through their recurrent connections, similar to the correlation-measuring rule used in [29]. With the inhibitory population, this rule acts to de-correlate responses and receptive fields.

### 4.8 Homeostasis

The STDP rules that lead to the network structure and selectivity in this study are separate from the homeostatic processes, which maintain stability and balance. Biological homeostasis typically operates on a slower timescale of hours or days ([69,70]). If only STDP and Hebbian plasticity were present, this would lead to pathological runaway dynamics ([71]). Therefore, there is a requirement for compensatory and stabilizing synaptic processes in computational models ([72]), typically implemented by homeostatic synaptic processes ([70]). In the network, there are two homeostatic processes: weight normalization ([73]), and an adaptive spiking threshold ([27]), which can be likened to activity-dependent synaptic scaling and intrinsic plasticity respectively ([73–75]). Both of these homeostatic mechanisms directly ensure that the weights and the firing rates, respectively, remain stable. In the study, these processes were found to be sufficient for network stability and function: when the mean weight values for the different connection groups were jittered randomly, or when the total number of neurons were scaled up, the network behaviour remained robust to perturbations of the network parameters, and the network was still able to learn the diverse range of V1 RFs.

### 4.9 The use of natural images during training

It was important to use natural images to train the model because it is known from sparse coding models that the efficient code for natural images is the Gabor function receptive fields observed in the visual cortex ([3]). Images with different statistics such as gratings or white noise would be expected to result in different receptive fields.

It is true that there are weak orientation maps observed in mammals soon after birth, and these cannot be due to learning from visual features ([76]). Cortical neurons likely derive this weak form of orientation tuning from waves of retinal activity known to occur in pre-natal mammals ([77]). However, the fully tuned Gabor functions that form the sparse code for natural scenes are not observed at this early age ([78]).

### 4.10 Future work

In this study, current-based synapses were used, where synaptic inputs were treated as currents directly injected into the neuron. However, using conductance-based synapses would be more biologically realistic. Conductance-based synapses would allow the effective time constant to change to maintain excitatory and inhibitory balance over a wide range of firing rates ([49]). Consequently, this may affect temporal and population sparseness.

The network included a lateral excitatory-excitatory pathway, which was given a low strength. The functional role of lateral excitatory connections in the brain remains unclear but is likely to be associated with the representation of temporal features of visual information that were not explored in this study. Increased weight of excitatory-excitatory connections can easily lead to pathological spiking. The use of spatiotemporal stimulus, such as natural video is therefore an important aspect of future work. Additionally, introducing biological distance-dependent spatial connectivity may allow for the emergence of cortical maps such as orientation maps.

The biological network explored in this study could form the basis for an artificial spiking neural network that is possible to implement in neuromorphic hardware.

## Supporting information

S1 Supplementary information Network with weights initialized to low values learned biological receptive fields. (PDF)

S1 Fig Receptive fields of excitatory and inhibitory neurons when small weights are initialized: (i) Excitatory receptive fields of 64 randomly chosen neurons. (ii) Inhibitory receptive fields of 25 randomly chosen neurons. Each box is a receptive field of a neuron where red represents ON and blue represents OFF which have values normalized. Neural parameters as described in Table 3.
(TIFF)

S2 Supplementary information Baseline firing before and after learning. (PDF)

S2 Fig Network response to spontaneous input: Response of output neurons is plotted when input neurons have a constant firing rate (i) with initialized weights before learning and (ii) after learning with natural images with spiking thresholds set back to the same level as before learning. Mean (black line) and standard deviation (blue for excitatory and red for inhibitory neurons) are plotted. Neural parameters as described in Table 3.
(TIFF)

S3 Supplementary information The effect of target rate on sparseness. (PDF)

S3 Fig Spike rate and sparseness: (i) Output spike rate of a LIF neuron as a function of excitatory Poisson input rate. The spike-threshold has been adjusted to produce a mean spike rate across all inputs of 1, 10, or 100 Hz (ii) The same data normalized to the target spike rates with the sparseness metric now shown in the legend. (iii) The resulting calculated sparseness values as a function of the target rate.
(TIFF)
