# Supplementary material for: Emergence of sparse coding, balance and decorrelation from a biologically-grounded spiking neural network model of learning in the primary visual cortex
Source: PLoS Comput Biol. 2025 Nov 21;21(11):e1013644. doi: 10.1371/journal.pcbi.1013644 (PMC12716757; doi:10.1371/journal.pcbi.1013644)
Supplement: S1 Supplementary information — (PDF) [file pcbi.1013644.s001.pdf]

S1. Network with weights initialized to low values learned biological receptive fields

The simulations presented in the main text had the weights initialized to the L1 norm upper bound. This section address the question of whether the network is able to learn appropriate receptive fields when the weights are initialized to low values. The weights are initialized as described in Methods 4.6, with one difference: the initial L1 norm of the weights was set to 1. After learning, the L1 norms of the weights reached their respective L1 norm upper bounds, and appropriate receptive fields are observed as seen in Fig Ai for excitatory neurons and Fig Aii for inhibitory neurons. These receptive fields are qualitatively similar to the receptive fields in the main text (Fig 4).

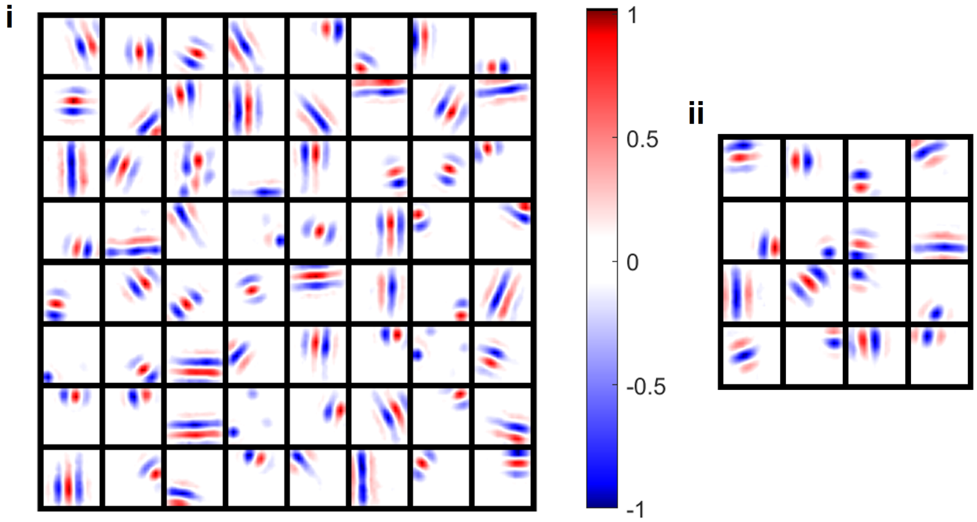

Figure A: **Receptive Fields of Excitatory and Inhibitory Neurons when Small Weights are Initialized:** (i) Excitatory receptive fields of 64 randomly chosen neurons. (ii) Inhibitory receptive fields of 25 randomly chosen neurons. Each box is a receptive field of a neuron where red represents ON and blue represents OFF which have values normalized. Neural parameters as described in Table 3.
