# Supplementary figures and images for: Emergence of sparse coding, balance and decorrelation from a biologically-grounded spiking neural network model of learning in the primary visual cortex
Source: PLoS Comput Biol. 2025 Nov 21;21(11):e1013644. doi: 10.1371/journal.pcbi.1013644 (PMC12716757; doi:10.1371/journal.pcbi.1013644)

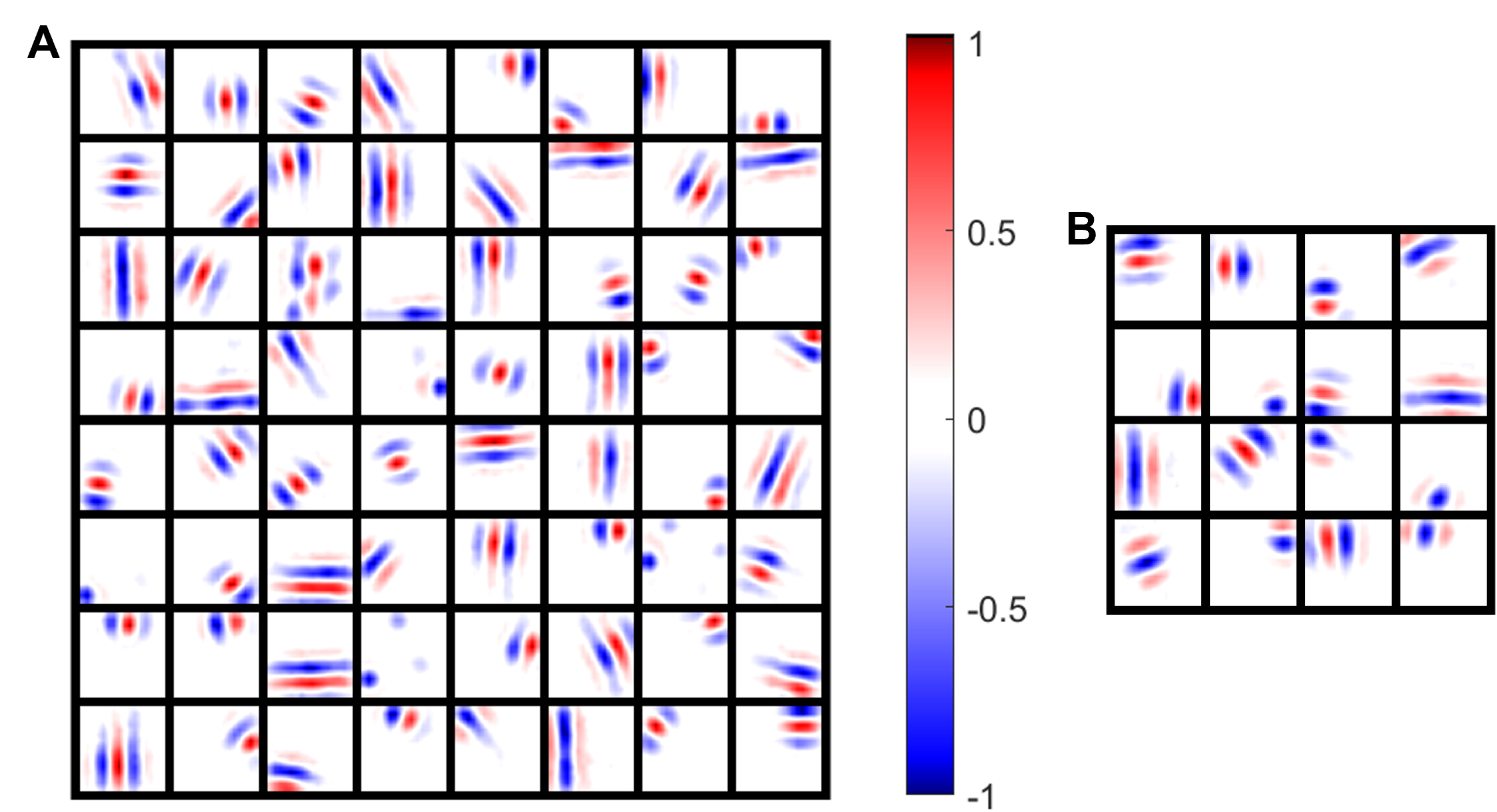

Supplement: S1 Fig — (ii) Inhibitory receptive fields of 25 randomly chosen neurons. Each box is a receptive field of a neuron where red represents ON and blue represents OFF which have values normalized. Neural parameters as described in Table 3. (TIFF) [file pcbi.1013644.s002.tif]

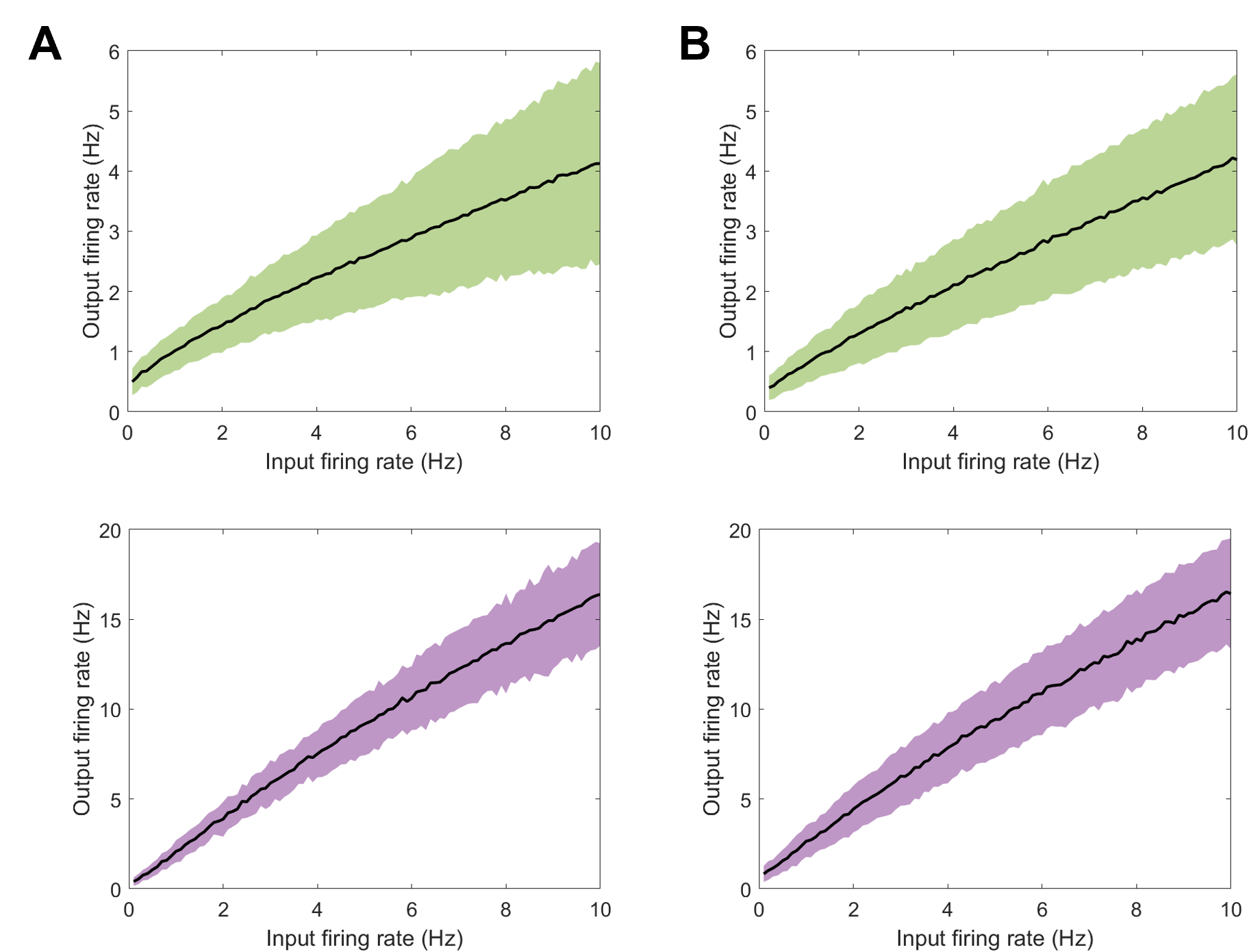

Supplement: S2 Fig — Mean (black line) and standard deviation (blue for excitatory and red for inhibitory neurons) are plotted. Neural parameters as described in Table 3. (TIFF) [file pcbi.1013644.s004.tif]

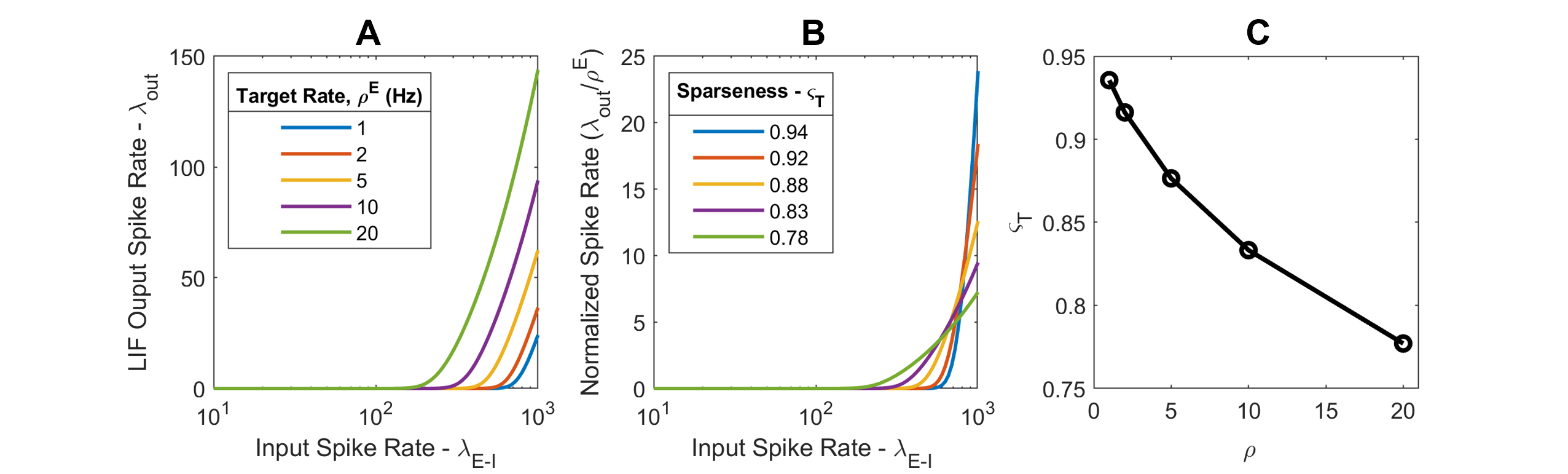

Supplement: S3 Fig — The spike-threshold has been adjusted to produce a mean spike rate across all inputs of 1, 10, or 100 Hz (ii) The same data normalized to the target spike rates with the sparseness metric now shown in the legend. (iii) The resulting calculated sparseness values as a function of the target rate. (TIFF) [file pcbi.1013644.s006.tif]
