# Supplementary material for: Emergence of sparse coding, balance and decorrelation from a biologically-grounded spiking neural network model of learning in the primary visual cortex
Source: PLoS Comput Biol. 2025 Nov 21;21(11):e1013644. doi: 10.1371/journal.pcbi.1013644 (PMC12716757; doi:10.1371/journal.pcbi.1013644)
Supplement: S2 Supplementary information — (PDF) [file pcbi.1013644.s003.pdf]

S2. Baseline firing before and after learning

Before presenting natural images, the weights were kept fixed and the input neurons fired at a background rate of 1 Hz. The spiking thresholds were allowed to adapt and the target firing rates of the excitatory and inhibitory neurons were set to $\rho_0^{\mathrm{E}} = 1$ Hz and $\rho_0^{\mathrm{I}} = 2$ Hz respectively. This represents the resting state of the network before learning, where all neurons fire with a spontaneous activity. Following this, the target firing rates become $\rho^{\mathrm{E}} = 2$ Hz and $\rho^{\mathrm{I}} = 4$ Hz respectively when presented with static visual stimuli.

The synaptic weights in the network of spiking neurons described here changed due to learning with static image stimuli as described in Subsection 4.4. Neural circuits need to maintain plasticity to accommodate changes in connectivity and synaptic strength during development and learning. Also important is maintaining the spontaneous firing of neurons before and after learning for the stability and function of neural circuits. However, it is a challenge to maintain both ongoing plasticity and stability at the same time (Schulz, 2006). Baseline activity is maintained throughout the learning process as a result of synaptic scaling implemented by L1 weight normalisation (as described in Section 4.4.3).

Prior to the learning process, when the network is presented with background firing rates, where all input neurons fire at the same spontaneous rates, the output neurons fire at some level proportional to this input rate. After the learning process using natural images, when the spiking thresholds were returned to the levels before learning through a homeostatic mechanism for the output firing rate (Turrigiano, 2012), the input-output response curve appears to be similar before and after learning, as shown by comparing Fig Bi with Fig Bii. However, the input-output response curve before learning is determined by the initial conditions and the method of weight initialization, while the input-out response curve after learning is determined by the learnt weights that underwent plasticity.

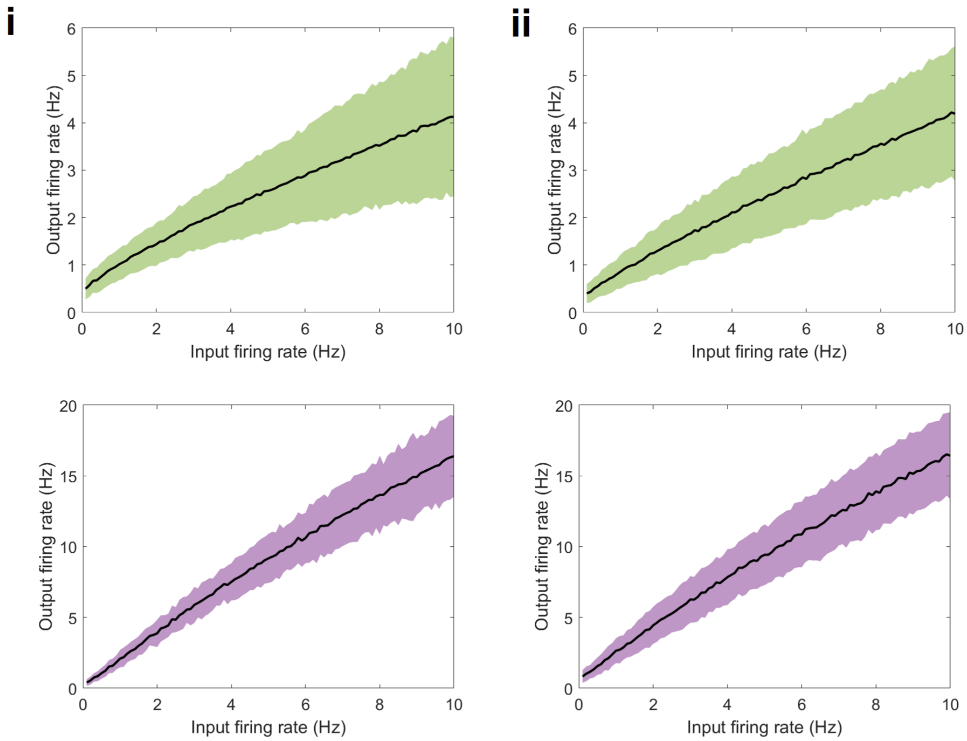

Figure B: **Network Response to Spontaneous Input:** Response of output neurons is plotted when input neurons have a constant firing rate (i) with initialized weights before learning and (ii) after learning with natural images with spiking thresholds set back to the same level as before learning. Mean (black line) and standard deviation (blue for excitatory and red for inhibitory neurons) are plotted. Neural parameters as described in Table 3.
