# Supplementary material for: Emergence of sparse coding, balance and decorrelation from a biologically-grounded spiking neural network model of learning in the primary visual cortex
Source: PLoS Comput Biol. 2025 Nov 21;21(11):e1013644. doi: 10.1371/journal.pcbi.1013644 (PMC12716757; doi:10.1371/journal.pcbi.1013644)
Supplement: S3 Supplementary information — (PDF) [file pcbi.1013644.s005.pdf]

S3. The effect of Target Rate on Sparseness

To show why temporal sparseness (ς_{T}) decreases with increasing target spike rate (ρ^{E}) it is possible to use Siegert's formula for spike rate of a LIF neuron (Burkitt, 2006b):

$$\lambda_{\mathrm{out}} = \left\{ \tau_{\mathrm{r}} + \frac{\tau_{\mathrm{m}}}{\sigma_{\mathrm{Q}}} \sqrt{\frac{\pi}{2}} \int_{V_{\mathrm{rest}}}^{\theta} \mathrm{d}u \, \exp\left[\frac{(u-\mu_{\mathrm{Q}})^2}{2\sigma_{\mathrm{Q}}^2} \right] \left[1 + \mathrm{erf}\left(\frac{u-\mu_{\mathrm{Q}}}{\sigma_{\mathrm{Q}}\sqrt{2}} \right) \right] \right\}^{-1}, \tag{1}$$

where τ_r is the absolute refractory period, τ_{m} is the membrane time constant, V_{rest} is the reset membrane voltage and θ is the spike threshold voltage. μ_{Q} and σ_{Q} are the mean and variance of the free membrane potential with δ-current input from an excitatory Poisson process and inhibitory Poisson process:

$$\mu_{\mathrm{Q}} = \tau_{\mathrm{m}}(a_{\mathrm{E}}\lambda_{\mathrm{E}} - a_{\mathrm{I}}\lambda_{\mathrm{I}}) = \tau_{\mathrm{m}}\lambda_{\mathrm{E-I}}, \tag{2}$$

$$\sigma_{\mathrm{Q}} = \frac{\tau_{\mathrm{m}}}{2}(a_{\mathrm{E}}^2\lambda_{\mathrm{E}} - a_{\mathrm{I}}^2\lambda_{\mathrm{I}}) = \sqrt{\frac{\tau_{\mathrm{m}}\lambda_{\mathrm{E-I}}}{2}}, \tag{3}$$

where a_{E} is the amplitude of the excitatory input synapse, and λ_{E} and λ_{I} are the excitatory and inhibitory Poisson input rates. The simplified formulations of equations 2 and 3 are derived by substituting $a_{\mathrm{E}} = a_{\mathrm{I}} = 1$ and $\lambda_{\mathrm{E-I}} = \lambda_{\mathrm{E}} - \lambda_{\mathrm{I}}$.

Using equation 1 with $a_{\mathrm{E}} = 1$, $\tau_{\mathrm{m}} = 10$ ms, $\tau_{\mathrm{r}} = 1$ ms, and a reset voltage $V_{\mathrm{rest}} = 0$ the output rate was calculated for 100 input rates ($\lambda_{\mathrm{E-I}}$) logarithmically spaced between 10 Hz and 1000 Hz. This is a simulation of the statistics across images of the high-weight input synapses. The value of θ was adjusted until the average output rate ($\overline{\lambda_{\mathrm{out}}}$) across all these inputs was 1, 10, or 100 Hz. The results are shown in Fig Ci.

It is clear that a lower target spike rate, associated with a higher threshold, gives lower spike rates in response to the range of input spike rates. However, by plotting these rates as a multiple of the mean it can be seen that the lower target rate appears to give a significantly more sparse distribution of output rates (Fig Cii). This is confirmed using the Treves-Rolls sparseness metric (Equation 16). The LIF neuron with a threhsold adjusted to achieve a 1 Hz mean output rate gave a value of $\varsigma_T = 0.94$ while the 10 Hz mean output rate resulted in $\varsigma_T = 0.59$. Plotting these values results in a curve (Fig Ciii) that can be compared to the trend observed in the full spiking model (Fig 10B).

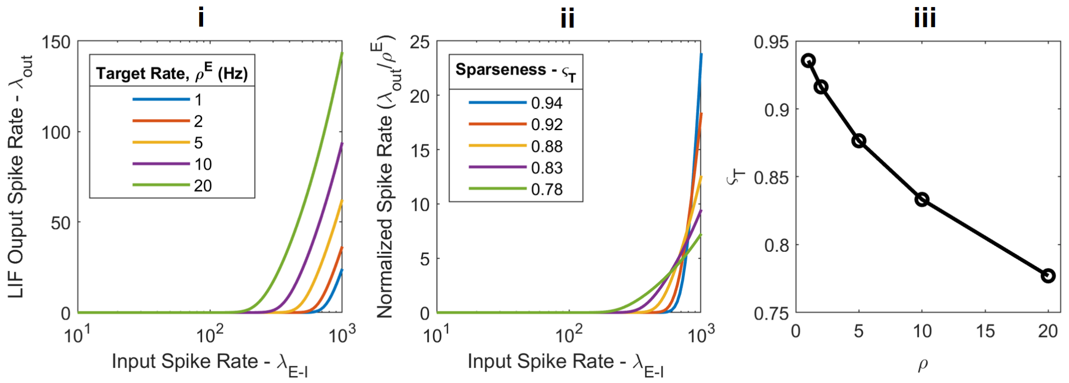

Figure C: **Spike Rate and Sparseness**: (i) Output spike rate of a LIF neuron as a function of excitatory Poisson input rate. The spike-threshold has been adjusted to produce a mean spike rate across all inputs of 1, 10, or 100 Hz (ii) The same data normalized to the target spike rates with the sparseness metric now shown in the legend. (iii) The resulting calculated sparseness values as a function of the target rate.
